# The effectiveness of digital cognitive intervention in patients with traumatic brain injury: systematic review and meta-analysis

**DOI:** 10.3389/fneur.2025.1651443

**Published:** 2025-10-07

**Authors:** Kejia Chi, Jiangfeng Chen, Shiwei Zhou, Zheqi Han

**Affiliations:** Department of Emergency, Shaoxing People’s Hospital, Shaoxing, Zhejiang, China

**Keywords:** digital cognitive intervention, computerized cognitive intervention, virtual reality based cognitive intervention, cognition function, traumatic brain injury, meta-analysis

## Abstract

**Objective:**

This meta-analysis aims to quantitatively evaluate the effects of digital cognitive intervention (non-immersive computer- and immersive virtual reality (VR)-based) on cognitive function and psychosocial outcomes in patients with traumatic brain injury (TBI), and to explore potential moderating factors.

**Methods:**

A systematic search was conducted in PubMed, the Cochrane Library, Embase, and Web of Science databases from their inception to April 3, 2025. Standardized mean differences (SMDs) and 95% confidence intervals (CIs) were calculated to estimate effect sizes, and heterogeneity was assessed using the I^2^ statistic.

**Results:**

A total of 16 studies were included; 9 employed computer-based cognitive interventions and 7 used VR-based interventions. The results showed that both types of interventions significantly improved global cognitive function (SMD: 0.64, 95% CI: 0.44 to 0.85, I^2^ = 0%), executive function (SMD: 0.32, 95% CI: 0.17 to 0.47, I^2^ = 15%), attention (SMD: 0.40, 95% CI: 0.02 to 0.78, I^2^ = 0%) and social cognitive function (SMD: 0.46, 95% CI: 0.20 to 0.72, I^2^ = 0%) in TBI patients. However, no significant improvements were observed in memory, processing speed, activities of daily living, or psychosocial outcomes (self-efficacy, anxiety/depression). Subgroup analysis indicated that VR-based interventions were more effective than traditional cognitive therapy. Moreover, VR interventions had a positive effect on depression in TBI patients. A greater number of training sessions may further enhance cognitive benefits.

**Conclusion:**

This meta-analysis supports the efficacy of digital cognitive intervention in improving cognitive function in TBI patients. We recommend individualized treatment programs to more effectively address cognitive impairments.

## 1 Introduction

Traumatic brain injury (TBI) refers to focal or diffuse neurological damage resulting from various external mechanical forces, such as impact, rapid acceleration or deceleration, or penetrating injury (1). TBI affects an estimated 55 million people worldwide each year and remains a leading cause of injury-related mortality and long-term physical and cognitive disability, particularly among young adults (2–5). Given its substantial health and socioeconomic burden, TBI represents a major public health challenge (2). Patients with TBI commonly experience motor, cognitive, and affective impairments that significantly impact their quality of life and that of their caregivers alike (6). Among these, cognitive deficits are particularly concerning, as they hinder rehabilitation outcomes by limiting work capacity, social functioning, and independence in daily activities, thereby compounding the overall disease burden (7, 8).

Cognitive rehabilitation (CR) for TBI aims to restore impaired cognitive functions and develop compensatory strategies to improve daily functioning (9, 10). Over the past three decades, growing evidence has demonstrated that CR can significantly enhance functional outcomes in patients with TBI through both the recovery of lost abilities and the implementation of adaptive strategies (11–14). However, the practical delivery of CR presents notable challenges. These interventions often require substantial time, resources, and financial commitment, which can lead to reduced engagement and poor adherence to treatment protocols, ultimately diminishing their therapeutic effectiveness (15).

In recent years, technological advancements have enabled the development of innovative rehabilitation approaches, such as digital intervention including computer-assisted therapies and virtual reality (VR) interventions (16–18). These methods have demonstrated clinical effectiveness in treating various cognitive disorders, such as those associated with stroke and dementia (19–21). Conventional cognitive rehabilitation typically involves therapist-led interventions, delivered either individually or in group settings, often with support from family members or multidisciplinary teams (22). Technology-mediated interventions (e.g., VR, computer-based training) are increasingly being adopted as viable alternatives to traditional face-to-face cognitive therapy due to their multiple advantages (23).

Computer-based cognitive intervention (CCI) employs digital tools to enhance or restore cognitive functions, including memory, attention, problem-solving, and work-related skills, through targeted exercises and adaptive training programs (20). The potential benefits of CCI include ease of self-administration, improved accessibility, and greater cost-effectiveness (24). Additionally, CCI can boost participant engagement through varied formats (e.g., videos, gamification), unlimited responsiveness, and adaptive feedback, promoting a sense of interactivity and enjoyment (25). Most CCI programs are delivered through digital platforms or mobile applications, enhancing flexibility and convenience-especially for those with mobility challenges or limited access to rehabilitation services-thus significantly improving care accessibility (24). Traditional CCI mainly presented on a standard two-dimensional desktop or laptop monitor. Interaction is typically achieved via mouse, keyboard, or touchscreen. While delivered via computer, these programs offer a limited sense of immersion and presence.

VR is an emerging computer-based technology that provides users with dynamic, three-dimensional simulated environments in which they can interact as if in real physical spaces (26). It primarily employs head-mounted displays that provide a wide field of view and head-tracking, creating a strong sense of presence and immersion. The potential advantages of VR include: (1) dynamically adjusting stimulus intensity and task difficulty based on patient performance, (2) integrating cognitive and functional training to promote neuroplastic recovery, and (3) enabling objective quantification of user behavior and performance metrics (27, 28). As a result, VR enhances training specificity and patient engagement by reducing boredom and frustration through a more sophisticated and ecologically valid methodology (29). VR has demonstrated efficacy as both an assessment and therapeutic tool for addressing motor impairments and cognitive dysfunction, including executive function and functional activities (30, 31).

A systematic review by Alashram (32) reported that computerized cognitive training (CCT) improved various cognitive domains in patients with TBI and that combining CCT with other interventions produced greater benefits than CCT alone. However, the findings were qualitative rather than quantitative, and the review focused solely on CCT, excluding newer technologies such as VR. Several studies employing VR have shown that it can enhance cognitive and behavioral functioning in patients with TBI (16, 17). A review by Andrei et al. (33) further indicated that VR-based interventions significantly improved cognitive functions in TBI patients, particularly in the domains of attention, executive function, and visuospatial abilities. Nonetheless, current evidence supporting the use of VR in cognitive neurorehabilitation for TBI remains limited, and there is no clear clinical consensus regarding its therapeutic efficacy (16). To the best of our knowledge, no meta-analysis has yet examined the effects of digital (computer- and VR-based) cognitive intervention in TBI. Therefore, we conducted a meta-analysis to quantitatively assess the impact of these interventions on cognitive function and to further investigate differences in therapeutic efficacy and contributing factors when compared to passive controls and traditional rehabilitation methods.

## 2 Methods

This meta-analysis was conducted in strict accordance with the Preferred Reporting Items for Systematic Reviews and Meta-Analyses (PRISMA) guidelines (34).

### 2.1 Search strategy

A systematic literature search was performed using the PubMed, Cochrane Library, Embase, and Web of Science databases to identify studies published from inception to April 3, 2025. The following Medical Subject Headings (MeSH) and keyword terms were used in combination: (traumatic brain injury) AND (cognitive intervention OR cognitive training OR cognitive therapy OR cognitive rehabilitation) AND (computerized OR virtual reality). Search terms within each thematic group were combined using “OR,” and the thematic groups were then combined using “AND.” The full search strategies are detailed in Supplementary Table S1. Additionally, the reference lists of relevant articles were manually screened to identify any additional eligible studies. Two authors (KJC and JFC) independently performed the literature search and screening. Any disagreements were resolved through discussion with a third author (ZQH).

### 2.2 Study selection

Eligible articles were selected based on strict adherence to the PICOS framework (Population, Intervention, Comparison, Outcome, Study design). Inclusion criteria were as follows: (1) population: individuals with a confirmed diagnosis of TBI; (2) intervention: digital cognitive intervention delivered using computer-based platforms or VR; (3) comparison: control conditions including active controls (participants engaged in non-structured interventions), passive controls (participants who received no intervention or were placed on waitlists), usual care, or traditional cognitive interventions; (4) outcome: at least one reported outcome related to cognitive function—either global cognition or specific cognitive domains (e.g., executive function, memory, processing speed, social cognition, attention), neuropsychiatric symptoms (e.g., anxiety, depression), activities of daily living (ADL), or self-efficacy; and (5) study design: randomized controlled trials (RCTs) or quasi-RCTs. Exclusion criteria included: (1) publications in languages other than English; (2) studies involving participants with other neurological conditions (e.g., ischemic or hemorrhagic stroke); and (3) studies without full-text access or with unavailable primary outcome data.

### 2.3 Data extraction

Two authors (KJC and JFC) independently extracted the experimental details and outcome data. Any discrepancies were resolved through discussion with a third reviewer (ZQH) until consensus was reached. For each included study, the following information was extracted: first author, year of publication, country, participant characteristics (sex, age, sample size, diagnosis, and baseline cognitive function), intervention design for both the treatment group (including details of the cognitive intervention, session length, frequency, and total duration) and the control group, as well as outcome measures. Primary outcomes included global and domain-specific cognitive functions (executive function, memory, processing speed, social cognitive function, and attention). Secondary outcomes included anxiety, depression, activities of daily living, and self-efficacy. The measurement tools used for each outcome variable are listed in Supplementary Table S2.

### 2.4 Quality assessment

Two authors independently assessed the risk of bias for each included study using the Cochrane Collaboration’s Risk of Bias Tool (35). The tool evaluates six domains: selection bias, performance bias, detection bias, attrition bias, reporting bias, and other potential sources of bias. Each domain was rated as having a ‘low,’ ‘high,’ or ‘unclear’ risk of bias. In cases of disagreement or uncertainty, a third investigator (SWZ) was consulted to reach a final decision.

### 2.5 Statistical analyses

Means, standard deviations, and sample sizes were extracted or calculated from the included studies. Hedges’ g, a variation of Cohen’s d, was used to compute standardized mean differences (SMDs) as a measure of between-group effect size (ES). For each outcome variable, the ES was reported along with its corresponding 95% confidence interval (CI). When studies reported multiple measures for a given cognitive domain, data from all relevant comparisons were extracted and assessed. For cognitive tests where higher scores indicated poorer performance, effect sizes were reversed to ensure that all positive values consistently reflected improvements in cognitive function. Effect sizes of 0.2, 0.5, and 0.8 were interpreted as small, moderate, and large, respectively. A *p*-value < 0.05 was considered statistically significant. A random-effects model was applied due to anticipated methodological and clinical heterogeneity in treatment effects across studies (36).

Heterogeneity was assessed using the I^2^ statistic and Cochran’s Q test. I^2^ values of 25–50%, 50–75%, and ≥75% were interpreted as indicating low, moderate, and high heterogeneity, respectively (37). For Cochran’s Q test, a *p*-value < 0.10 was considered indicative of significant heterogeneity (37). Sensitivity analyses were performed to examine the robustness of the results. Subgroup analyses were conducted to identify potential factors influencing outcomes and to further investigate sources of heterogeneity. All statistical analyses were conducted using Review Manager (RevMan) software version 5.3 (The Cochrane Collaboration) and Stata version 16 (StataCorp LP, College Station, TX, United States).

## 3 Results

### 3.1 Identification of studies

**Figure 1:**
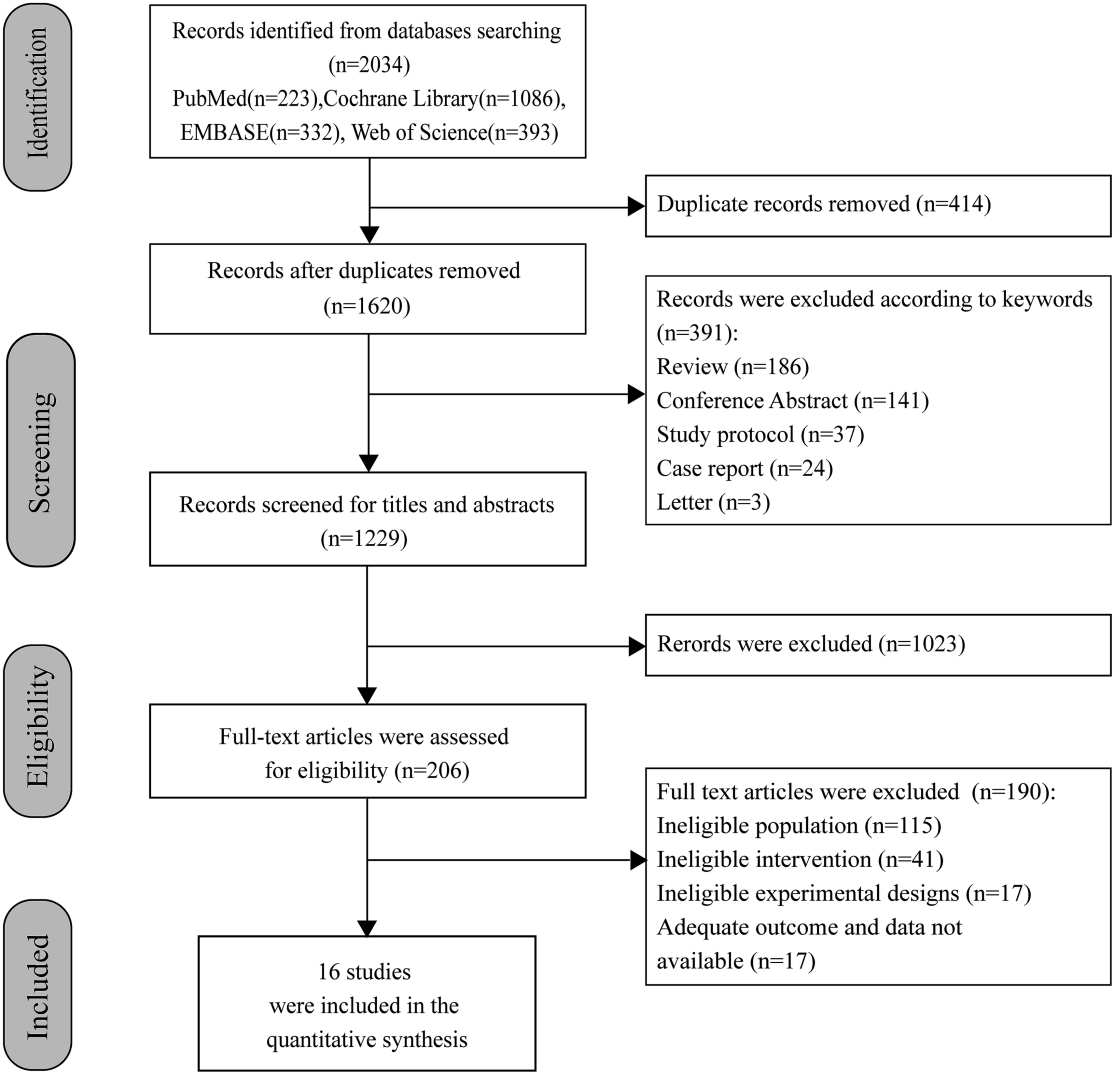
Flow diagram of literature identification and selection process.

The study selection process and reasons for exclusion are illustrated in Figure 1. A total of 2,034 records were identified through database searches. After removing duplicate records (*n* = 414), excluding studies based on keywords (*n* = 391) and screening titles and abstracts (*n* = 1,023), 206 full-text articles were retrieved for further evaluation. Of these, 190 articles were excluded for the following reasons: ineligible population (*n* = 115), ineligible intervention (*n* = 41), ineligible experimental design (*n* = 17), and lack of available outcome data (*n* = 17). Ultimately, 16 studies were included in this meta-analysis.

### 3.2 Characteristics of the included studies

The characteristics of the 16 included trials are summarized in Table 1. Collectively, these studies involved 720 patients with TBI. Half of the studies (*n* = 8) utilized non-immersive computer-based cognitive interventions, seven employed VR-based cognitive interventions (VR-CI), and one study used a gaming-based rehabilitation approach. The duration of interventions ranged from a single session to 13 weeks. The included studies covered TBI cases of varying severity, from mild to severe. In terms of participant demographics, 14 studies focused on adult patients, while the remaining two involved pediatric participants. Regarding time since injury, seven studies included both subacute and chronic TBI patients, three focused exclusively on subacute cases, five on chronic cases, and one study did not report the time since injury. Included studies involved different types of control groups. Active control groups (*n* = 5) received alternative interventions not expected to influence the targeted outcomes. Passive control groups (*n* = 4) consisted of participants who received no intervention or were placed on a waiting list. Usual care control groups (*n* = 1) provided standard rehabilitation services (e.g., physical therapy) without the experimental cognitive intervention. Conventional cognitive intervention groups (*n* = 6) received traditional, non-digital cognitive therapy, typically delivered through in-person sessions with therapists.

**Table 1:** Characteristics of included studies.

The first author (Year)	Country	Mean age (SD)	Patients	Time since injury	Intervention	Control group	Duration	Frequency	Follow-up time	Outcomes
P Rodríguez-Rajo (2024) (52)	Spain	39.87 (15.54)	Moderate or severe TBI	73.02 days (sub-acute)	CCT-social cognition (GNPT)	Active control: CCT-nonsocial cognition	7 weeks (21 sessions)	60 min/session, 3 sessions/ week	/	Social cognition
Tobias Lohaus (2024) (67)	Germany	44.95 (16.70)	TBI	>3 months	CCI (SoCoBo online)	Active control: CCI (RehaCom)	12 weeks (48 sessions)	30 min/session, 4 times/week	/	Social cognition
Sing-Fai Tam (2004) (59)	China	18–45 years	closed head injury	>3 months	CCT (self-paced, feedback, personalized and visual presentation)	No intervention	2 weeks (10 sessions)	20-30 min/session, 5 times/week	/	Everyday memory
Henry W Mahncke (2021) (68)	USA	33.8 (8.7)	Mild TBI	>3 months	CCT (BrainHQ)	Active control (computer games)	13 weeks (65 sessions)	60 min/session, 5 sessions /week	3 months	Global cognitive function, TIADL, depression
Hei-Fen Hwang (2020) (50)	China	66.95 (11.12)	TBI	3.75 ± 4.89 months	CCT (RehaCom)	Usual care	6 months	At least 45 min/session, 1 session/week	6 months	Global cognitive function (multiple domains), ADL, depression
Maritta Välimäki (2018) (49)	Finland	40.67 (12.19)	TBI	>12 months	rehabilitation gaming (CogniFit)	No intervention	8 weeks	At least 30 min/day	3 months	cognitive function (multiple domains), depression, Self efficacy
Gerald T Voelbel (2021) (51)	USA	44.52 (12.71)	TBI	>12 months	CCT (Brain Fitness Program)	No intervention	12 weeks (40 sessions)	60 min/session, 3–4 sessions/week	/	cognitive function (multiple domains), depression, anxiety
Mark L Ettenhofer (2019) (28)	USA	52 (8.97)	TBI	>12 months	VR driving rehabilitation (NeuroDRIVE)	Wait List	4 weeks (6 sessions)	90 min/session	/	cognitive function (multiple domains), depression,
David Wai Kwong Man (2013) (26)	China	18–55 years	Mild to moderate TBI	4 ± 8.58 months	AI VR based vocational training (AIVTS)	Conventional vocational training (PEVTS)	1 months (12 session)	20–25 min/session	/	Executive function
Rosaria De Luca (2023) (69)	Italy	44.6 (16.13)	Moderate to severe TBI	>6 months	VR-CT	Conventional CT	8 weeks (24 sessions)	60 min/session, (3 sessions/week)	/	Global cognitive function, executive function
Andrew J Darr (2024) (24)	USA	31.16 (7.92)	closed mild TBI	>3 months	CCT (Lumosity or UCR Brain Games)	Traditional CR	4 weeks (12 sessions)	60 min/session, 3 sessions/week	/	cognitive function (multiple domains)
Michele Jacoby (2013) (27)	Israel	29.25 (12.69)	TBI	113 ± 62.85 days	VR-CT (Virtual Mall)	Standard CT	(10 session)	45 min/ session, 3–4 sessions/week	/	executive function
Rosaria De Luca (2019) (44)	Italy	39.93 (10.1)	Mild to moderate TBI	3 to 6 months	VR-CT (BTs Nirvana)	Traditional CR	8 weeks (24 sessions)	60 min/session, 3 sessions/week	/	Global cognitive function (multiple domains), depression, anxiety
Rosaria De Luca (2022) (55)	Italy	43.56 (16.04)	Severe TBI	>3 months	VR-CT (VRRS)	Conventional CT	8 weeks (24 sessions)	45 min/ session, 3 sessions/week	/	Global cognitive function (multiple domains), depression,
Jiabin Shen (2022) (38)	USA	12.96 (3.27)	TBI	NA	VR-based interactive CT	Active control: VR game without CT	20–30 min	At least 1 training session, about 20–30 min	2 months	Global cognitive function
Nikita Tuli Sood (2024) (46)	Australia	10.62 (2.89)	Mild to severe TBI	>6 months	CCT (Cogmed)	Active-control (Lexia) with no WM memory training	5 weeks	50 min/day, 5 days/week	6 months	WM

ADL, activities of daily living; AI, artificial intelligent; AIVTS, artificial intelligent virtual reality-based vocational training system; CCI, computer-based cognitive intervention; CCT, computerized cognitive training; CI, cognitive intervention; CR, cognitive rehabilitation; CT, cognitive training; GNPT®, the Guttmann Tele-Rehabilitation Platform, NeuroPersonalTrainer®; NeuroDRIVE, Neurocognitive Driving Rehabilitation in Virtual Environments; PEVTS, psycho-educational vocational training program; SoCoBo, the Treatment Program for Deficits in Social Cognition and Social Competencies of the Ruhr University Bochum; TBI, traumatic brain injury; TIADL, timed instrumental activities of daily living; VR, virtual reality; VR-CT, virtual reality -based cognitive train; VRRS, virtual reality rehabilitation system; WM, working memory.

### 3.3 Study quality

**Figure 2:**
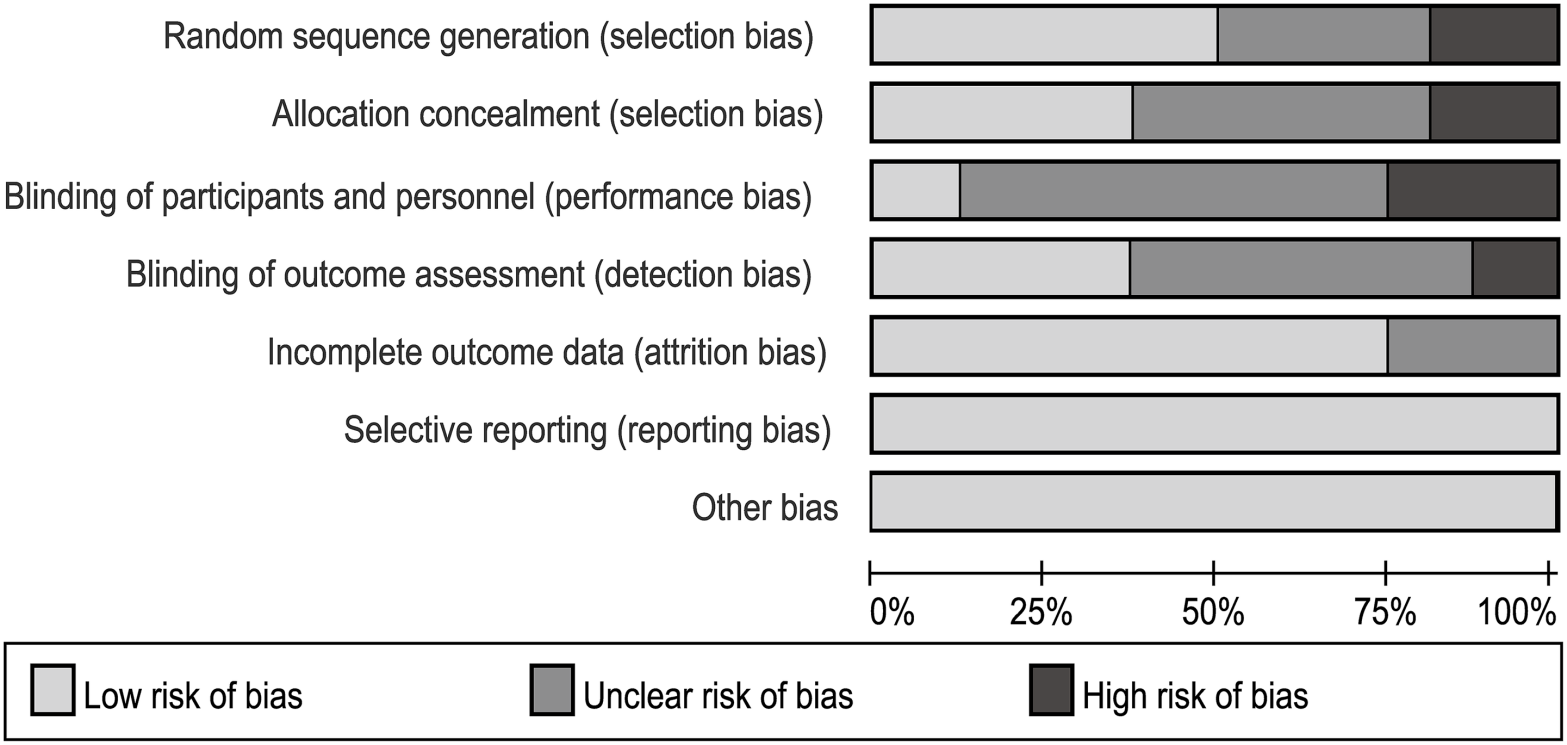
Risk-of-bias assessments of the included studies based on the Cochrane collaboration tool.

The comprehensive results of the risk of bias assessment are shown in Figure 2. Of the 16 included studies, eight (50%) explicitly described their methods of randomization, five (31.3%) did not specify the randomization procedures used, and three (18.8%) employed quasi-randomization for group allocation. Due to the nature of the interventions and practical limitations, the majority of studies did not implement or report blinding procedures for participants, personnel, or outcome assessors. Four studies were assessed as having an unclear risk of attrition bias due to insufficient information regarding the reasons for participant withdrawal. In terms of selective reporting and other sources of bias, all studies were rated as having a low risk. Overall, no serious concerns were identified regarding the risk of bias across the included studies.

### 3.4 Results of the meta-analyses

#### 3.4.1 Global cognitive function

**Figure 3:**
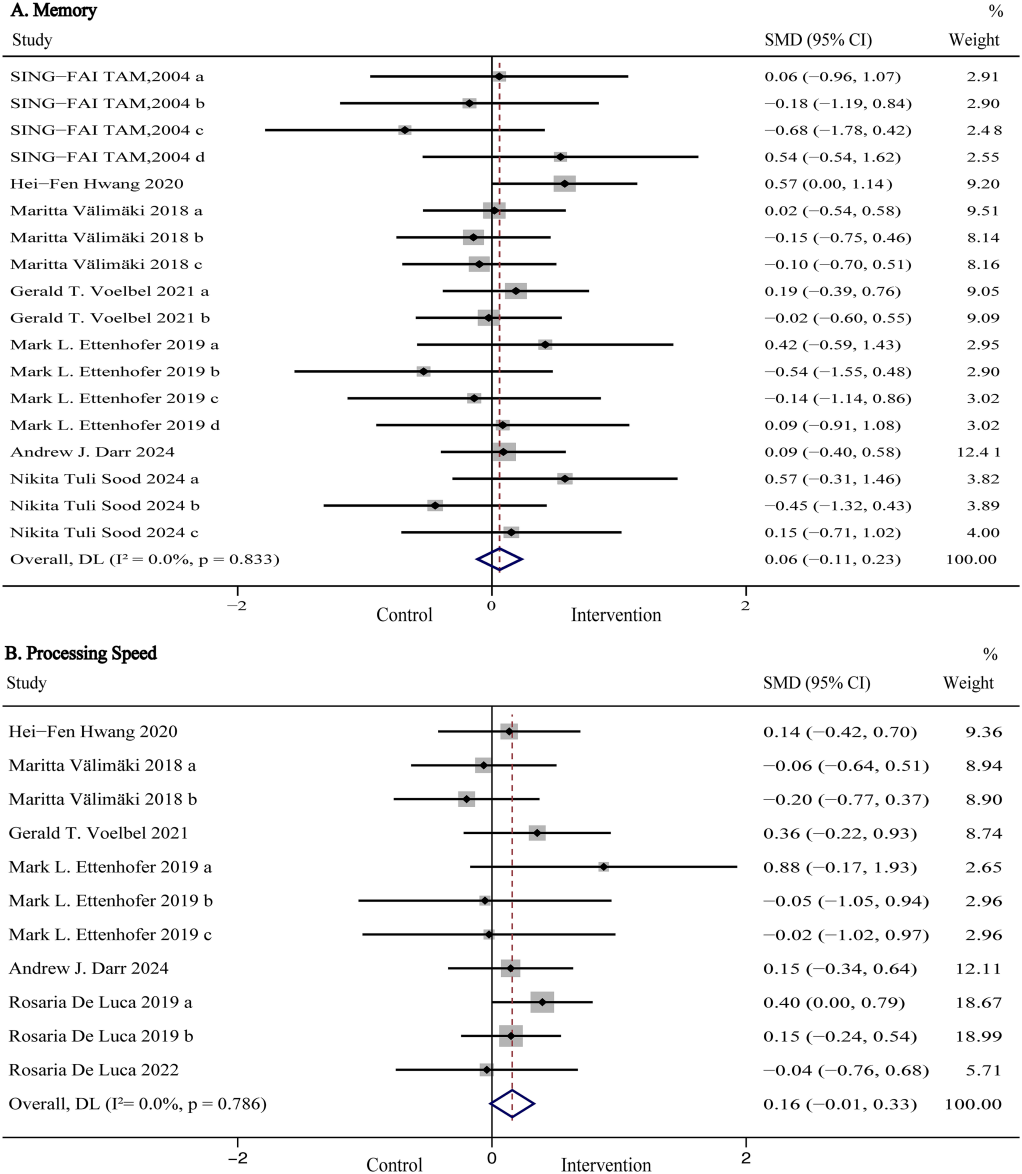
Forest plot of the digital cognitive intervention on **(A)** global cognitive function and **(B)** executive function in patients with traumatic brain injury. CI, confidence interval; SMD, standardized mean difference.

**Figure 4:**
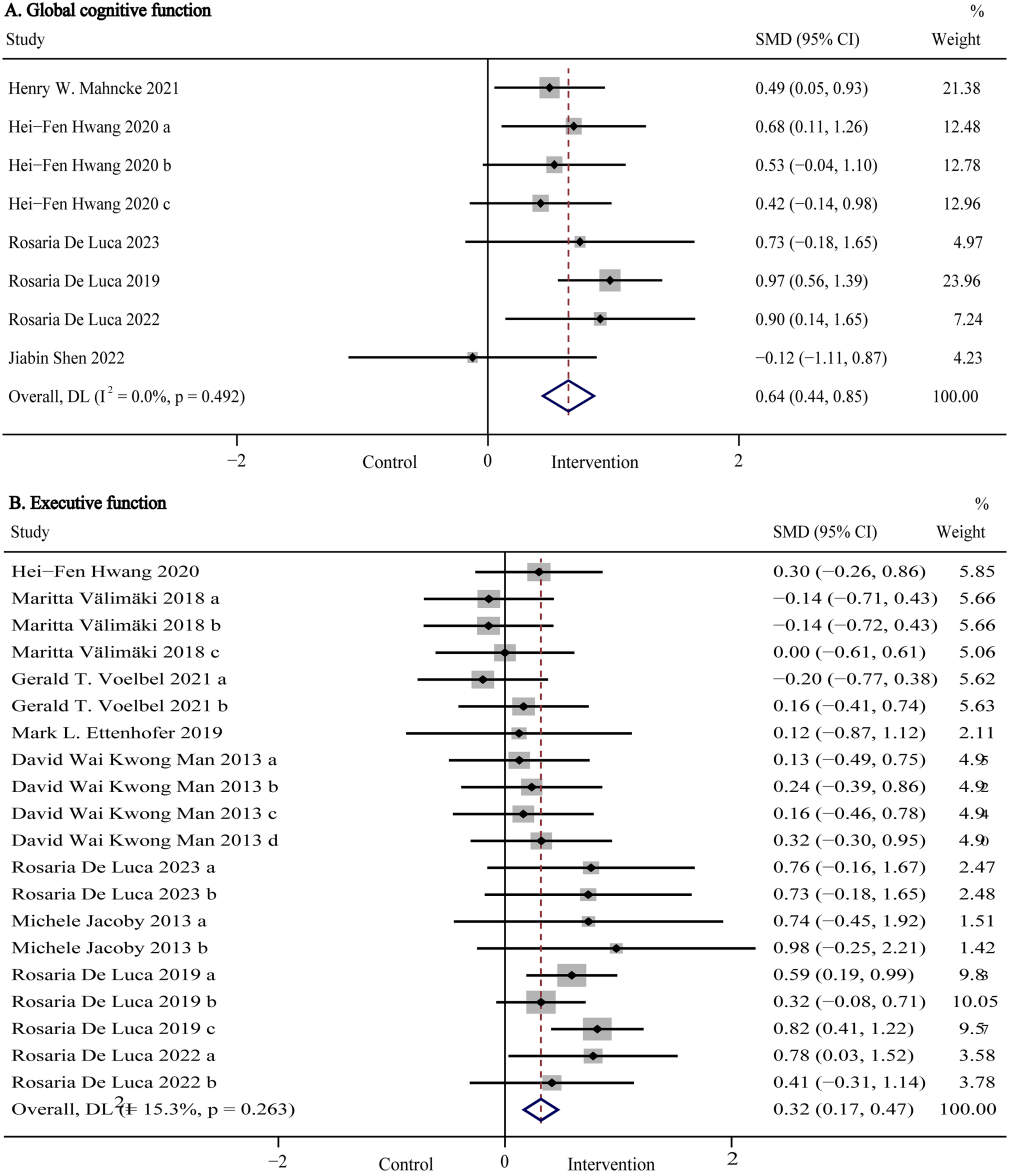
Forest plot of the subgroup analysis of computer- and VR-based cognitive intervention on **(A)** global cognitive function and **(B)** executive function according to intervention and control types. CCT, computerized cognitive training; CI, cognitive intervention; CI, confidence interval; SMD, standardized mean difference; VR, virtual reality.

Global cognitive function, representing a composite measure of overall cognitive performance, was evaluated in six studies (*n* = 864) using standardized assessments such as the Montreal Cognitive Assessment (MoCA), Mini-Mental State Examination (MMSE), Mattis Dementia Rating Scale (MDRS), Telephone Interview for Cognitive Status-Modified (TICS-M), and NIH Toolbox Composite Score. Pooled results are presented in Figure 3A and Table 2. Our meta-analysis showed that digital (computer- and VR-based) cognitive intervention significantly improved global cognitive function in patients with TBI (SMD:0.64, 95% CI: 0.44 to 0.85, *p* < 0.001; I^2^ = 0%). As shown in Figure 4A, subgroup analyses were conducted by stratifying intervention types and control group categories. The forest plot demonstrated that non-immersive CCI significantly improved global cognitive function compared to both usual care and active control groups (SMD: 0.52, 95% CI: 0.26 to 0.79, *p* < 0.001; I^2^ = 0%). Additionally, VR-CI demonstrated greater efficacy than conventional face-to-face cognitive rehabilitation (SMD: 0.92, 95% CI: 0.59 to 1.26, *p* < 0.001; I^2^ = 0%).

**Table 2:** Results of subgroup analysis.

Outcome	Comparisons (n)	Sample size	SMD with 95% CI	*p* value	I^2^(%)
Global cognitive function	8	398	0.64 [0.44, 0.85]	**<0.001**	0
Intervention type					
VR	4	166	0.76 [0.35, 1.17]	**<0.001**	26
Computer	4	232	0.52 [0.26, 0.79]	**<0.001**	0
Control type					
Active and usual care	5	248	0.48 [0.23, 0.74]	**<0.001**	0
Conventional CI	3	150	0.92 [0.59, 1.26]	**<0.001**	0
Participants					
Adult	7	382	0.68 [0.47, 0.88]	**<0.001**	0
Underage	1	15	−0.12 [−1.11, 0.87]	0.81	/
Session number					
<20 sessions	1	15	−0.12 [−1.11, 0.87]	0.81	/
≥20 sessions	7	382	0.68 [0.47, 0.88]	**<0.001**	0
Executive function	20	882	0.32 [0.17, 0.47]	**<0.001**	15
Intervention type					
VR	14	601	0.48 [0.32, 0.65]	**<0.001**	0
Computer	3	146	0.09 [−0.24, 0.42]	0.58	0
Games	3	135	−0.10 [−0.44, 0.24]	0.56	0
Control type					
Active and usual care	7	298	0.01 [−0.22, 0.24]	0.96	0
Conventional CI	13	584	0.49 [0.33, 0.66]	**<0.001**	0
Session number					
<20 sessions	7	201	0.27 [−0.01,0.55]	0.06	0
≥20 sessions	13	681	0.32[0.12, 0.53]	**0.002**	40
Memory	18	533	0.06 [−0.11, 0.23]	0.50	0
Intervention type					
VR	4	68	−0.04 [−0.54, 0.46]	0.87	0
Computer	11	332	0.13 [−0.09, 0.35]	0.23	0
Games	3	133	−0.07 [−0.41, 0.27]	0.69	0
Control type					
Active and usual care	17	468	0.06 [−0.13, 0.24]	0.56	0
Conventional CI	1	65	0.09 [−0.40, 0.58]	0.72	/
Memory domains					
Everyday Memory	4	58	−0.06 [−0.59, 0.46]	0.82	0
Working memory	10	374	0.04 [−0.16, 0.25]	0.68	0
Processing Speed	11	538	0.16 [−0.01, 0.33]	0.07	0
Intervention type					
VR	6	281	0.23 [−0.00, 0.47]	0.05	0
Computer	3	163	0.20 [−0.11, 0.52]	0.20	0
Games	2	94	−0.13 [−0.54, 0.27]	0.52	0
Control type					
Active and usual care	7	243	0.09 [−0.16, 0.35]	0.48	0
Conventional CI	4	295	0.21 [−0.02, 0.44]	0.07	0
Social cognitive function	6	244	0.46 [0.20, 0.72]	**<0.001**	0
Attention	3	110	0.40 [0.02, 0.78]	**0.04**	0
Anxiety	2	148	−0.31 [−0.76, 0.14]	0.18	43
Depression	7	372	−0.19 [−0.49, 0.10]	0.20	47
Intervention type					
VR	3	147	−0.64 [−0.97, −0.31]	**<0.001**	0
Computer	2	132	0.05 [−0.29, 0.39]	0.78	0
Games	1	45	0.03 [−0.56, 0.61]	0.92	/
Activity of daily living	3	149	−0.18 [−0.62, 0.26]	0.42	35
Self-efficacy	**5**	103	0.12 [−0.27, 0.51]	0.55	0

CI, cognitive function; VR, virtual reality; SMD, standardized mean difference. The bold values represent those with statistical significance (*p* < 0.05).

Further subgroup analyses were conducted to examine potential moderators by isolating one variable at a time, with results summarized in Table 2. The analyses indicated that digital cognitive intervention was significantly more effective in adult TBI patients (SMD: 0.68, 95% CI: 0.47 to 0.88, *p* < 0.001; I^2^ = 0%) compared to minors, although only one study involved underage participants (38). Moreover, interventions comprising more than 20 sessions showed superior efficacy (SMD: 0.68, 95% CI: 0.47 to 0.88, *p* < 0.001; I^2^ = 0%) compared to shorter protocols (<20 sessions), though again, only one study assessed interventions with fewer than 20 sessions (38). As such, these comparisons should be interpreted with caution.

#### 3.4.2 Executive function

Executive function refers to a set of higher-order cognitive processes, including working memory, cognitive flexibility, and inhibitory control, which regulate goal-directed behavior (39). Nine studies (*n* = 882) evaluated executive function using various standardized tools, including the Trail Making Test Part B (TMT-B), Wisconsin Card Sorting Test (WCST), Multiple Errands Test-Simplified Version (MET-SV), Executive Function Performance Test (EFPT), Simon Task, Behavior Rating Inventory of Executive Functioning-Adult version (BRIEF-A), Tower of London, and the Frontal Assessment Battery (FAB). Pooled results are presented in Figure 3B and Table 2. The meta-analysis showed that digital cognitive interventions significantly improved executive function in TBI patients (SMD: 0.32, 95% CI: 0.17 to 0.47, *p* < 0.001; I^2^ = 15%). As shown in Figure 4B, further subgroup analyses based on intervention type and control group indicated that VR-CI significantly enhanced executive function compared to conventional face-to-face cognitive therapy (SMD: 0.49, 95% CI: 0.33 to 0.66 *p* < 0.001; I^2^ = 0%). However, neither computer-based nor game-based cognitive interventions showed significant improvements in executive function compared to usual care or passive control groups. Additional subgroup analysis results related to executive function are summarized in Table 2. Consistent with findings from the global cognitive function subgroup, interventions involving more than 20 sessions demonstrated greater efficacy (SMD: 0.32, 95% CI: 0.12 to 0.53, *p* < 0.001; I^2^ = 40%) than shorter interventions (<20 sessions).

#### 3.4.3 Memory

**Figure 5:**
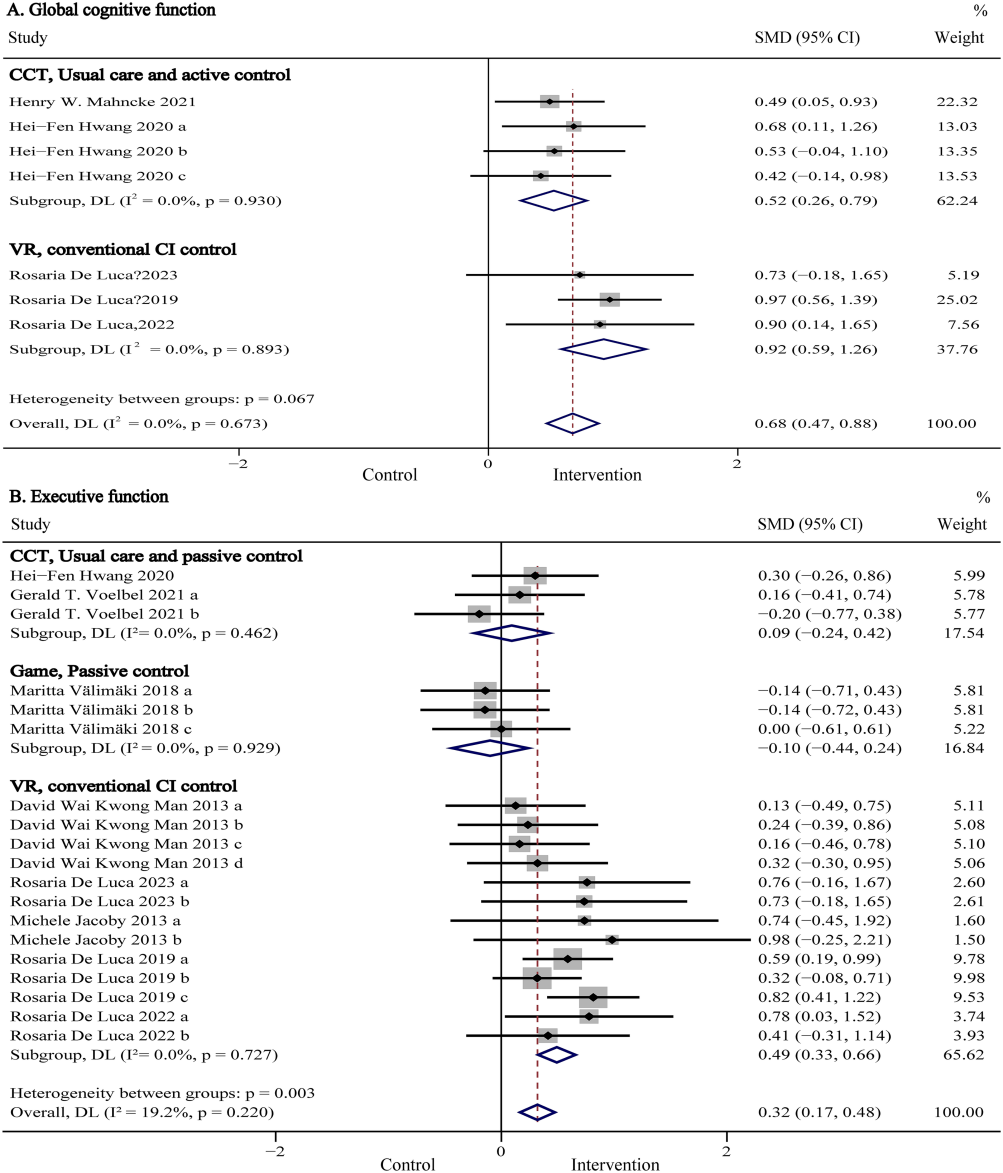
Forest plot of the digital cognitive intervention on **(A)** memory and **(B)** processing speed in patients with traumatic brain injury. CI, confidence interval; SMD, standardized mean difference.

Memory was assessed in seven studies (*n* = 533) using the MDRS-Memory, the Paced Auditory Serial Addition Test (PASAT), Rivermead Behavioral Memory Test (RBMT), California Verbal Learning Test-Second Edition (CVLT-II), and the Digit Span subtest of the Wechsler Adult Intelligence Scale IV (WAIS-IV). Pooled and subgroup analysis results are shown in Figure 5A and Table 2. The pooled results indicated that digital cognitive intervention did not produce a statistically significant improvement in memory among TBI patients (SMD: 0.06, 95% CI: −0.11 to 0.23, *p* = 0.500; I^2^ = 0%). Moreover, subgroup analyses revealed that variations in intervention type, control group, or memory domain had no significant influence on the primary outcome.

#### 3.4.4 Processing speed

Processing speed was assessed in seven studies (*n* = 538) using the Trail Making Test Part A (TMT-A), Symbol Digit Modalities Test (SDMT), WAIS-IV Symbol Search, WAIS-IV Coding, and Visual Search tasks. Pooled and subgroup analysis results are presented in Figure 5B and Table 2. The pooled results indicated that digital cognitive intervention had no statistically significant effect on processing speed in TBI patients (SMD: 0.16, 95% CI: −0.11 to 0.33, *p* = 0.070; I^2^ = 0%). Furthermore, subgroup analyses based on intervention type and control group classification revealed no significant effects on the primary outcomes.

#### 3.4.5 Social cognitive function

**Figure 6:**
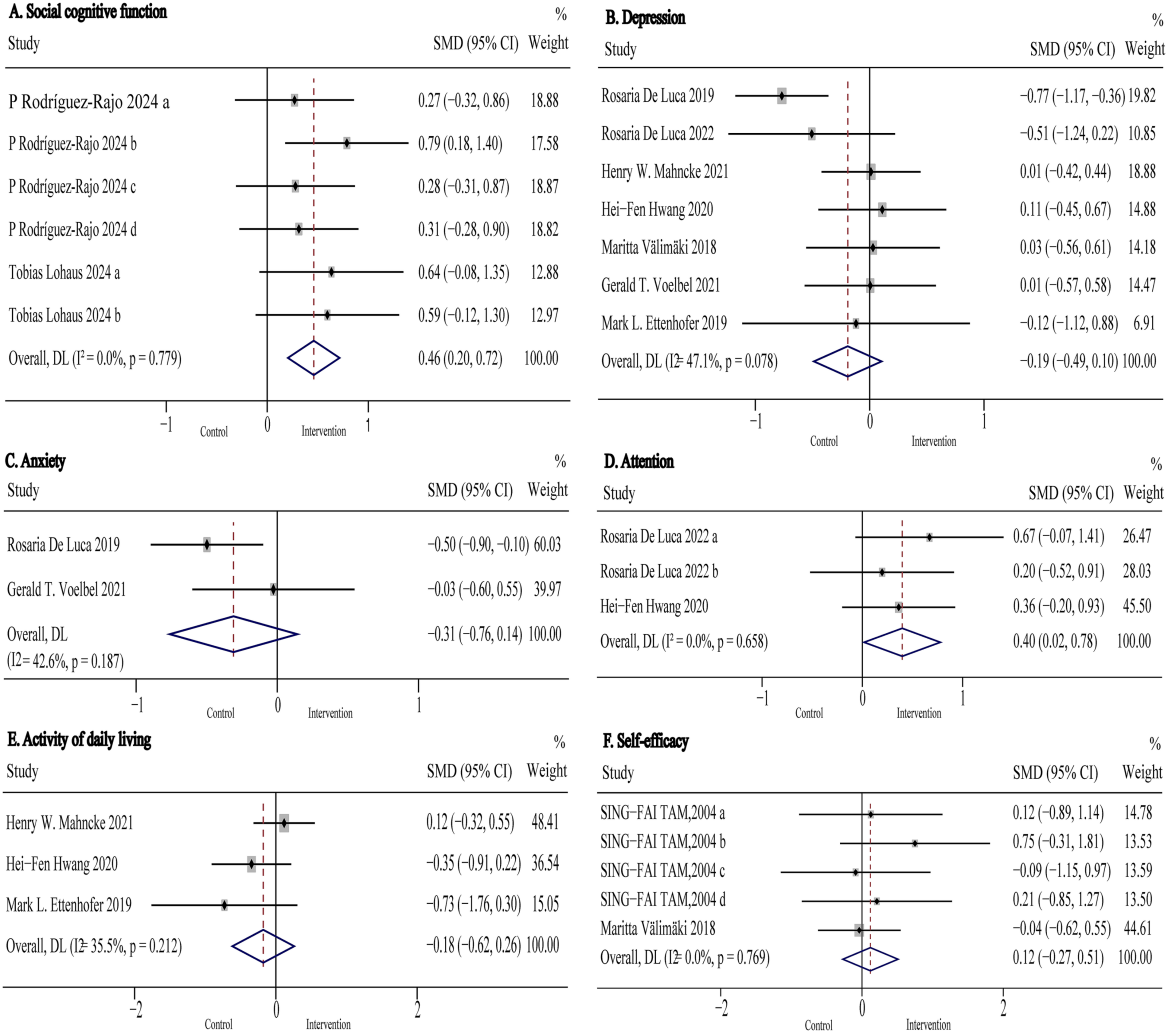
Forest plot of the digital cognitive intervention on **(A)** social cognitive function, **(B)** depression **(C)** anxiety, **(D)** attention, **(E)** activity of daily living and **(F)** self-efficacy in patients with traumatic brain injury. CI, confidence interval; SMD, standardized mean difference.

Social cognitive function was assessed in two studies (*n* = 244) using the Social Decision-Making Task (SDMT), Reading the Mind in the Eyes Test (RMET), Moving Shapes Paradigm (MSP), Facial Expressions of Emotion-Stimuli and Tests (FEEST), Emotion Recognition Index (ERI), and the German version of the Interpersonal Reactivity Index (IRI). Pooled results are shown in Figure 6A and Table 2. The analysis indicated that non-immersive CCI significantly improved social cognitive function compared to active control conditions in TBI patients (SMD: 0.46, 95% CI: 0.20 to 0.72, *p* < 0.001; I^2^ = 0%). Subgroup analyses were not conducted due to the limited number of studies evaluating outcomes in this domain.

#### 3.4.6 Attention

Attention was assessed in two studies (*n* = 110) using Attentive Matrices test (AMT), MoCA-Attention, and the Attention subscale of the MDRS-Attention. Pooled results are presented in Figure 6D and Table 2. The analysis showed that digital cognitive intervention significantly improved attention in TBI patients (SMD: 0.40, 95% CI: 0.02 to 0.78, *p* = 0.040; I^2^ = 0%). As with social cognitive function, subgroup analyses were not performed due to the small number of included studies.

#### 3.4.7 Anxiety and depression

Anxiety was assessed in two studies (*n* = 148) using the Beck Anxiety Inventory (BAI) and the Hamilton Rating Scale for Anxiety (HRS-A). Pooled results are presented in Figure 6C and Table 2. The analysis indicated that digital cognitive intervention had no significant effect on anxiety in TBI patients (SMD: −0.31 95% CI: −0.76 to 0.14, *p* = 0.18; I^2^ = 43%).

Depression was assessed in seven studies (*n* = 372) using the Hamilton Rating Scale for Depression (HRS-D), Beck Depression Inventory-II (BDI-II), Center for Epidemiologic Studies Depression Scale (CES-D), and the Patient Health Questionnaire-9 (PHQ-9). Pooled and subgroup analysis results are shown in Figure 6B and Table 2. The pooled results indicated that digital cognitive intervention had no significant overall effect on depression (SMD: −0.19 95% CI: −0.49 to 0.10, *p* = 0.20; I^2^ = 47%), indicating low to moderate heterogeneity. However, subgroup analysis revealed that VR-CI significantly reduced symptoms of depression in TBI patients (SMD: −0.64, 95% CI: −0.97 to −0.31, *p* < 0.001; I^2^ = 0%). In contrast, neither computer-based nor game-based cognitive interventions demonstrated statistically significant effects on depression when compared to usual care, active, or passive control groups (*p* > 0.05 for all comparisons).

#### 3.4.8 Activity of daily living and self-efficacy

Activity of daily living was assessed in three studies (*n* = 149) using the Timed Instrumental Activities of Daily Living (TIADL), Activities of Daily Living (ADL) scale, and the Physical Component Summary of the Medical Outcomes Study 36-Item Short-Form Health Survey (SF-36 PCS). Pooled results are presented in Figure 6E and Table 2. The analysis indicated that digital cognitive intervention had no significant effect on activities of daily living (SMD: −0.18 95% CI: −0.62 to 0.26, *p* = 0.42; I^2^ = 35%). Subgroup analysis was not conducted due to the limited number of included studies.

Self-efficacy was assessed in two studies (*n* = 103) using self-efficacy rating scales. Pooled results are shown in Figure 6F and Table 2. The analysis demonstrated that non-immersive CCI had no significant effect on self-efficacy (SMD: 0.12, 95% CI: −0.27 to 0.51, *p* = 0.55; I^2^ = 0%).

## 4 Discussion

Based on the current evidence reviewed, our results demonstrate that digital cognitive intervention significantly improved global cognitive function, executive function, attention, and social cognitive function in patients with TBI. However, no significant improvements were observed in memory, processing speed, ADL, or psychosocial outcomes, including self-efficacy and anxiety/depression. Subgroup analyses revealed that VR-CI resulted in significantly greater improvements in global cognitive and executive functions compared to conventional face-to-face cognitive therapy. Moreover, a higher number of training sessions appeared to enhance cognitive benefits. VR-CI was also found to have beneficial effects on depression in TBI patients.

The manifestation and severity of cognitive dysfunction following TBI are influenced by various factors, including the nature of the injury, psychosocial circumstances, and individual patient characteristics (32). Common cognitive deficits post-TBI include memory impairments (90%), attention difficulties (82%), and executive dysfunction (75%) (40). Digital cognitive intervention leverage technology to enhance cognitive performance through targeted, engaging training paradigms. Computerized programs (e.g., Cogmed, Lumosity) use adaptive tasks to strengthen specific domains such as working memory and attention by reinforcing neural pathways through repetitive and progressively challenging exercises (41). VR interventions immerse users in simulated real-world environments, facilitating multisensory integration and ecological transfer by simultaneously engaging memory, executive functions, and visuospatial skills (42). A fundamental mechanism underlying the effectiveness of these interventions is neuroplasticity—the brain’s capacity to reorganize and form new synaptic connections in response to structured therapeutic stimuli (43, 44). By capitalizing on this neuroadaptive potential, digital interventions can induce cognitive improvements in task-relevant brain regions (e.g., the prefrontal cortex for executive functions) through structured training (45). Additionally, their engaging interfaces and real-time feedback mechanisms enhance patient motivation and adherence, further supporting behavioral and cognitive recovery. Consistent with previous systematic reviews by Alashram (32) and Manivannan et al. (17), our meta-analysis found that digital cognitive intervention significantly enhanced global cognitive function, highlighting their therapeutic promise for cognitive rehabilitation. Subgroup analyses also revealed that patients who completed a greater number of training sessions showed superior cognitive gains. Therefore, a moderate increase in the number of training sessions may help optimize and sustain long-term cognitive benefits.

Two studies involving underage patients with TBI were included in our meta-analysis. Shen et al. found that the VR-CI program was safe for children with TBI and showed promising, though statistically non-significant, benefits for executive function (38). Another study reported that CCI significantly improved working memory in children with TBI, but this benefit was not sustained during the follow-up period (46). Notably, there are substantial developmental differences in cognition between children and adults. The ongoing neuroplasticity of the developing brain may enhance responsiveness to cognitive interventions (32). Therefore, further research is warranted to investigate the safety and efficacy of digital cognitive interventions in pediatric TBI populations.

Our subgroup analyses showed that VR-CI led to significantly greater improvements in global cognitive function and executive functioning compared to traditional face-to-face cognitive therapy. VR technology enables the creation of interactive, multisensory, three-dimensional environments that support comprehensive behavioral monitoring, offering clinical assessment and rehabilitation capabilities beyond those of conventional psychoeducational approaches (26). Executive function encompasses a range of high-level cognitive skills, including goal setting and initiation, planning and organization, task execution, and performance monitoring and regulation (47). These skills are essential for executing goal-directed behaviors and play a critical role in activities of daily living (ADLs). For example, Jacoby et al. employed a virtual reality supermarket to enhance executive function in TBI patients, demonstrating that VR-acquired skills successfully transferred to real-world tasks (27). The multisensory stimulation and interactive nature of VR environments allow patients to repeatedly practice tasks in contexts that closely resemble real-life situations, a benefit not achievable through conventional cognitive rehabilitation. As such, VR-CI represents a promising alternative approach for improving executive function in TBI patients.

Our results showed that CCI, including rehabilitation gaming, did not produce significant improvements in executive function compared to usual care or passive control groups. A previous systematic review reported that CCI could enhance executive function in patients with acquired brain injury, including TBI (48). However, since only three studies in our analysis examined the effects of CCI on executive function, these findings should be interpreted with caution due to the limited evidence base. One of the included studies on gaming-based rehabilitation noted that outcomes could have been influenced by factors such as the participants’ broad age range, variability in game types, their attitudes toward the therapy, among others (49). The computerized interventions in the remaining two studies appeared to primarily target improvements in attention and information processing (50, 51). These findings suggest that personalized CCI programs tailored to the specific cognitive deficits of individual patients may improve treatment efficacy by better addressing their unique therapeutic needs.

Social cognition is a critical domain for individuals with TBI, as it underpins social reintegration, emotional cue interpretation, and interpersonal relationships-functions commonly impaired post-injury (52). Traditional cognitive interventions tend to emphasize non-social cognitive domains such as memory and executive function, often neglecting aspects of social cognition (47, 53). Our findings showed that CCI significantly improved social cognitive function. Although only two studies included social cognition outcomes, their results provide preliminary evidence supporting the therapeutic potential of CCI in this area.

Enhancement of attention is crucial for both functional recovery and active engagement in rehabilitation programs (54). Our meta-analysis found a statistically significant improvement in attention following digital cognitive intervention. Although only two studies included attentional outcomes, their findings provide preliminary evidence supporting the effectiveness of these interventions in this cognitive domain. Therefore, additional high-quality studies are needed to further explore the impact of digital cognitive interventions on attention and social cognition. Several studies have also reported a negative correlation between reductions in depressive symptoms and improvements in attention (55, 56). Given that depressive symptoms are critical determinants of neurorehabilitation efficacy, addressing them is essential. VR environments can incorporate exposure therapy and relaxation exercises, which have been shown to reduce depressive symptoms, phobias, and post-traumatic stress (57). In line with this, our subgroup analysis demonstrated a meaningful reduction in depressive symptoms following VR-CI. However, no significant reduction in anxiety symptoms was observed following digital cognitive interventions. This may be partially due to the limited number of eligible studies included. Additionally, interventions targeting anxiety in TBI patients often involve computer-based cognitive behavioral therapy, which was beyond the scope of our review (58).

Our pooled results did not show significant improvements in memory or processing speed following digital cognitive intervention. This may be attributed to a lack of targeted training focused on these specific domains during the interventions. We extracted results from all memory-related scales used in the included studies, which covered a broad range of memory subtypes, such as working memory, everyday memory, short-term memory, and delayed memory. The varying effects of digital interventions on different memory domains could contribute to the heterogeneity of findings. Furthermore, the lack of sensitivity of some assessment tools may have limited the detection of subtle memory improvements associated with digital training (59). While our findings differ from several previous reviews that reported improvements in memory-including working memory-following digital cognitive interventions in patients with TBI (60, 61). However, both Rodríguez-Rajo et al. (52) and Phillips et al. (62) found no statistically significant differences in memory outcomes between intervention and control groups. Therefore, future research should utilize more sensitive and standardized assessment tools, develop targeted interventions, and conduct larger, high-quality randomized controlled trials to elucidate the efficacy of digital interventions in specific cognitive domains such as memory and processing speed.

No significant improvements in ADL were observed in this review, which may be attributed to the limited number of studies (*n* = 3) that provided analyzable data using ADL-related scales. Notably, none of these three studies incorporated specific ADL training tasks within their cognitive intervention programs. Prior evidence suggests a well-established association between improvements in executive function and attention and enhanced performance in ADL tasks (63). ADL is a critical domain, as one of the primary goals of CI is to support patients’ reintegration into real-world functional activities. Therefore, future CI studies should include standardized assessments of ADL to better evaluate functional outcomes. Self-efficacy, on the other hand, has demonstrated a significant independent effect on the efficacy of cognitive rehabilitation (59). Encouragement and positive feedback during therapy can enhance patients’ perceived self-efficacy. However, our findings showed no significant improvement in self-efficacy following computer-based cognitive interventions. The study by Tam et al. (59) found that self-efficacy was significantly enhanced in participants who received targeted feedback interventions. As self-efficacy plays a key role in brain injury rehabilitation, future cognitive intervention studies should routinely include standardized measures of self-efficacy and consider integrating motivational elements to support its improvement.

VR-CI has also been demonstrated to improve global cognitive function, executive function and memory among stroke patients compared to control treatments (21). Alashram et al. reported that the efficacy of CCI for visual and verbal working memory in acquired brain injury (ABI) population (64). ABI, encompassing etiologies such as stroke, hypoxic–ischemic injury, and brain tumors, shares with TBI the common endpoint of disrupted neural networks and consequent cognitive impairment. A core sequelae of ABI is the disruption of inherent neuroplasticity, the brain’s ability to reorganize itself by forming new neural connections. Both focal (e.g., stroke) and diffuse (e.g., TBI, anoxia) injuries can impair this process. CCI is fundamentally engineered to harness and drive neuroplasticity through the principles of massed practice and intensity. This intensive practice is believed to promote synaptic strengthening and efficiency within damaged or alternative neural networks (65). The game-like, engaging nature of many immersive VR-CI applications can increase adherence to the high repetitions needed for neuroplasticity (66). Therefore, the efficacy of digital cognitive interventions is not limited to TBI alone but is potentially generalizable to a wide range of ABI patients.

Our meta-analysis quantitatively confirmed the beneficial effects of digital cognitive intervention on cognitive function in patients with TBI. The low heterogeneity observed among the included studies enhances the robustness of the findings. However, this meta-analysis has several limitations. First, the results for some outcomes may have been influenced by the limited number of studies available. Subgroup analyses exploring potential moderating factors were also constrained by the small sample of studies. Several covariates may influence treatment effects, including: (a) the severity and age range of TBI patients (from mild to severe), (b) the time elapsed since injury, (c) the duration of follow-up after the intervention, and (d) the presence or absence of one-on-one coaching during treatment. Second, there is a lack of standardization in cognitive outcome assessments across studies, which underscores the need for more precise and consistent evaluation methods. Third, this review only included English-language publications, which may have introduced geographic and cultural bias. To better understand the effectiveness and influencing factors of digital cognitive intervention in TBI, larger, high-quality randomized controlled trials with more rigorous designs are urgently needed.

## 5 Conclusion and clinical recommendations

Our meta-analysis demonstrated that digital (computer- and VR-based) cognitive intervention have a positive impact on global cognitive function, executive function, attention, and social cognitive function in patients with TBI. However, these interventions did not show significant effects on memory, processing speed, ADL, or psychosocial outcomes (self-efficacy, anxiety, and depression). Subgroup analyses revealed that VR-CI were significantly more effective than traditional cognitive therapy in improving global cognitive and executive functions. VR-CI also showed beneficial effects on depressive symptoms. Moreover, a greater number of training sessions may enhance the cognitive benefits achieved.

In clinical practice, digital cognitive intervention is recommended to enhance cognitive functions in both adult and pediatric patients with TBI. Although more further researches are warranted to investigate the safety and efficacy of digital cognitive interventions in pediatric TBI populations. In global cognitive function and execution function, VR-CI demonstrate superior efficacy compared to conventional cognitive intervention. Given the variability in cognitive deficit profiles and intervention protocols focused on different specific domains, we recommend individualized treatment strategies to more effectively address cognitive impairments in TBI patients. Further research is needed to determine the most appropriate digital cognitive intervention programs tailored to individual patient characteristics.

## Data Availability

The original contributions presented in the study are included in the article/Supplementary material, further inquiries can be directed to the corresponding author.

## Glossary

ADL: activities of Daily Living
AMT: Attentive Matrices test
BAI: Beck Anxiety Inventory
BDI-II: Beck Depression Index II
BRIEF-A: Behavior Rating Inventory of Executive Functioning-Adult version
CCI: computer-based cognitive intervention
CCT: computerized cognitive training
CES-D: Center for Epidemiologic Studies Depression Scale
CI: confidence interval
CR: cognitive rehabilitation
CVLT-II: California Verbal Learning Test-II
EFPT: Executive Function Performance Test
ERI: Emotion Recognition Index
ES: effect size
FAB: Frontal Assessment Battery
FEEST: Facial Expressions of Emotion-Stimuli and Tests
HRS-A: Hamilton Rating Scale for Anxiety
HRS-D: Hamilton Rating Scale for Depression
IRI: German version of the Interpersonal Reactivity Index
MDRS: Mattis Dementia Rating Scale
MeSH: medical subject heading
MET-SV: Multiple Errands Test-Simplified Version
MMSE: Mini-mental state examination
MoCA: Montreal Cognitive Assessment
MSP: Moving Shapes Paradigm
PASAT: Paced Auditory Serial Addition Test
PHQ-9: Patient Health Questionnaire-9
PRISMA: Preferred Reporting Items for Systematic Reviews and Meta-Analyses
RBMT: Rivermead Behavioural Memory Test
RCT: randomized controlled trial
RMET: Reading the Mind in the Eyes Test
SDMT: Social Decision Making Task
SDMT: Symbol Digit Modalities Test
SF-36 PCS: Medical Outcomes Study 36-Item Short-Form Health Survey Physical Component Summary
SMD: standardized mean difference
TBI: traumatic brain injury
TIADL: Timed Instrumental Activities of Daily Living
TICS-M: Telephone Interview for Cognitive Status-Modified
TMT-A: Trail Making Test part A
TMT-B: Trail Making Test part B
VR: virtual reality
VR-CI: virtual reality-based cognitive intervention
WAIS-IV: Wechsler Adult Intelligence Scale IV
WCST: Wisconsin Card Sorting Test
